# Fast Orientation of Video Images of Buildings Acquired from a UAV without Stabilization

**DOI:** 10.3390/s16070951

**Published:** 2016-06-23

**Authors:** Michal Kedzierski, Paulina Delis

**Affiliations:** Department of Remote Sensing and Photogrammetry, Faculty of Civil Engineering and Geodesy, Military University of Technology, Warsaw 00908, Poland; michal.kedzierski@wat.edu.pl

**Keywords:** close range photogrammetry, collinearity equations, exterior orientation, image sequence, UAV

## Abstract

The aim of this research was to assess the possibility of conducting an absolute orientation procedure for video imagery, in which the external orientation for the first image was typical for aerial photogrammetry whereas the external orientation of the second was typical for terrestrial photogrammetry. Starting from the collinearity equations, assuming that the camera tilt angle is equal to 90°, a simplified mathematical model is proposed. The proposed method can be used to determine the X, Y, Z coordinates of points based on a set of collinearity equations of a pair of images. The use of simplified collinearity equations can considerably shorten the processing tine of image data from Unmanned Aerial Vehicles (UAVs), especially in low cost systems. The conducted experiments have shown that it is possible to carry out a complete photogrammetric project of an architectural structure using a camera tilted 85°–90° (φ or ω) and simplified collinearity equations. It is also concluded that there is a correlation between the speed of the UAV and the discrepancy between the established and actual camera tilt angles.

## 1. Introduction

The assurance of providing adequate protection of cultural heritage sites, as well as preserving their authenticity, can only be obtained by creating a comprehensive inventory of a given site. This includes the definition of the type, shape, dimensions and geospatial location of the given structure. One of the ways to create such an inventory for a cultural heritage building is based on its photogrammetric documentation in the form of a three-dimensional model.

Generating a 3D model of a historical structure using photogrammetric methods can be troublesome due to the height of the structure. Whether a camera is used or a terrestrial laser scanner, too great a height of the structure will greatly limit the possibility of using terrestrial photogrammetry techniques. In such instances, better results can be obtained by integrating terrestrial imagery data with data acquired from a low altitude flights. Using an Unmanned Aerial Vehicle (UAV) as a platform for the sensor ensures that relatively large scale imagery can be acquired, which warrants high-quality end products.

In recent years UAVs are being increasingly used in architectural photogrammetry, being a fundamental module of management and conservation of national cultural heritage sites [1]. Together with terrestrial laser scanning [2], imagery is the main source of information when producing cultural heritage inventory. The purpose of photogrammetric systems based on UAVs, in which the data acquisition module is a video camera, is to acquire data to develop orthophotomaps [3,4], digital terrain models [5,6,7], 3D city models [8], 3D models of buildings [9] and sculptures [10]. UAV systems prove particularly useful where the access to an architectural object is difficult, which may be due to the topography or close proximity to other architectural objects. The complexity of the shapes of buildings is also a frequent obstacle during the implementation of photogrammetric techniques. It is necessary to use a relative image orientation obtained from multiple camera positions or a terrestrial laser scanner. In the case of an insufficient number of camera positions, detection of tie points in the images may not be feasible. Then, it is very helpful to use a sequence of video images. Finding similarities in adjacent images is much easier than in images taken from distant camera positions [11]. In addition, in the case of video data, the redundant number of video images makes it possible to eliminate blurred images [12,13], images with a low radiometric quality index [14] or those, for which the values of the exterior orientation parameters significantly differ within an image sequence which may be a result of flight instability.

In the literature, one can often find descriptions of systems involving the integration of data from the two altitudes: terrestrial and aerial. These methods often relate to the integration of point clouds from terrestrial and aerial laser scanning [15], point clouds and imagery data [16] or image data from terrestrial and aerial levels. An example includes the studies conducted by Bolognesi et al. [17]. The authors have developed a 3D model of a historical architectural structure based on image data acquired using a Canon EOS M high resolution digital camera mounted on UAV platforms and from ground level. In studies conducted by Püschel et al. [18] a method was developed of documenting an architectural monument, consisting of the integration of terrestrial images with images captured using a UAV. The image data were recorded by a non-metric digital camera in video mode.

The large majority of video image sequence orientation methods are based on Structure from Motion (SFM) algorithms [19]. SFM relies on determining the 3D point coordinates and the camera projection matrix simultaneously, based on homologous points measured on a large number of images [20]. It should be mentioned that the Rodriquez matrix method can be used to solve the problem of nonlinearity models of absolute and relative orientation. One of the advantages this method is the lack of gimble effect [21].

To ensure high accuracy of orientation of the video images, methods of image orientation based on the fundamental equation of photogrammetry, i.e., the collinearity equation should be used [22].

**Figure 1:**
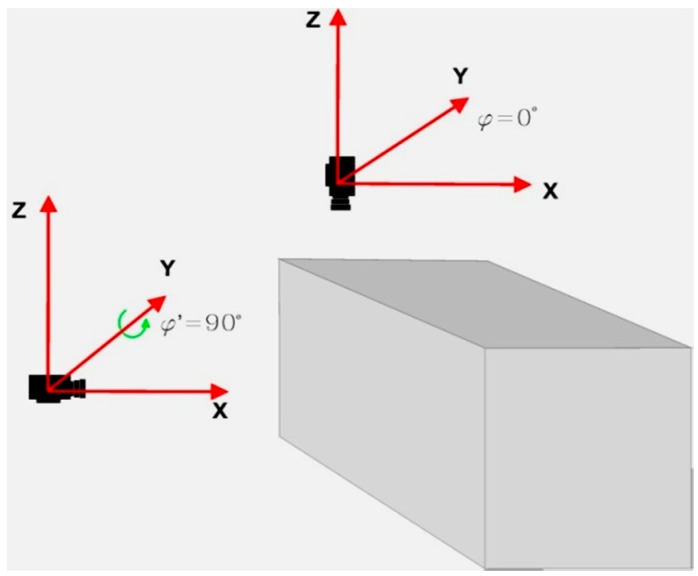
Transition of the video camera mounted on the UAV from aerial to terrestrial orientation.

This paper presents the issues which occur when performing an orientation of two images, of which one was acquired with a tilt angle close to 90°. Such a situation takes place quite often when conducting photogrammetric measurements of buildings based on video imagery. During such measurements, first the side walls of the building are filmed and then the camera is put into aerial orientation in order to acquire imagery from the roof. The aim of this research was to assess the possibility of conducting an orientation procedure of video imagery, in which the external orientation for the first image was typical for aerial photogrammetry whereas the external orientation of the second was typical for terrestrial photogrammetry. This type of orientation (Figure 1) of video images is a special case of processing two images for photogrammetric documentation of architectural structures. It is closely associated with the problem of integrating aerial and terrestrial imagery data. This issue is especially important, due to the fact that it occurs very often when conducting photogrammetric measurements of buildings. The problem of integrating aerial and terrestrial imagery data stems from using two different coordinate systems (and two rotation matrices): typical for aerial photogrammetry and for terrestrial photogrammetry in order to photogrametrically process both the walls and the roof of a given architectural structure in one unified coordinate system.

## 2. Proposed Method of Orientation for Terrestrial and Aerial Video Images

Assuming, that the axes of the terrestrial coordinate system are parallel to the building's walls, a rotation occurs about one of the axes of the terrestrial coordinate system X or Y (when the video camera makes the transition from aerial orientation to terrestrial orientation). In this case, the tilt angle (ω or φ) for the terrestrial image is close to 90°.

By adopting the rotation matrix and maintaining the order of the rotation ω φ κ [22]:

(1) $$A_{\omega \varphi \kappa }=\left[\begin{array}{ccc} a_{11} & a_{12} & a_{13}\\ a_{21} & a_{22} & a_{23}\\ a_{31} & a_{32} & a_{33} \end{array}\right]$$

The rotation matrix coefficient equations can be greatly simplified and take on the form shown in (Table 1):

By implementing the inverse of the collinearity equations for a stereo made up of video frames with two different orientations (aerial and terrestrial), the following set of equations was created:

(2) $$\left[\begin{array}{c} X=X_{0}^{\prime }+\left(Z-Z_{0}^{\prime }\right)\frac{\left(x^{\prime }-x_{0}\right){a^{\prime }}_{11}+\left(y^{\prime }-y_{0}\right){a^{\prime }}_{21}-{a^{\prime }}_{31\;}ck}{\left(x^{\prime }-x_{0}\right){a^{\prime }}_{13}+\left(y^{\prime }-y_{0}\right){a^{\prime }}_{23}-{a^{\prime }}_{33\;}ck}\;\\ Y=Y_{0}^{\prime }+\left(Z-Z_{0}^{\prime }\right)\frac{\left(x^{\prime }-x_{0}\right){a^{\prime }}_{12}+\left(y^{\prime }-y_{0}\right){a^{\prime }}_{22}-{a^{\prime }}_{32\;}ck}{\left(x^{\prime }-x_{0}\right){a^{\prime }}_{13}+\left(y^{\prime }-y_{0}\right){a^{\prime }}_{23}-{a^{\prime }}_{33\;}ck}\\ X=X_{0}^{\prime \prime }+\left(Z-Z_{0}^{\prime \prime }\right)\frac{\left(x^{\prime \prime }-x_{0}\right){a^{\prime \prime }}_{11}+\left(y^{\prime \prime }-y_{0}\right){a^{\prime \prime }}_{21}-{a^{\prime \prime }}_{31\;}ck}{\left(x^{\prime \prime }-x_{0}\right){a^{\prime \prime }}_{13}+\left(y^{\prime \prime }-y_{0}\right){a^{\prime \prime }}_{23}-{a^{\prime \prime }}_{33\;}ck}\\ Y=Y_{0}^{\prime \prime }+\left(Z-Z_{0}^{\prime \prime }\right)\frac{\left(x^{\prime \prime }-x_{0}\right){a^{\prime \prime }}_{12}+\left(y^{\prime \prime }-y_{0}\right){a^{\prime \prime }}_{22}-{a^{\prime \prime }}_{32\;}ck}{\left(x^{\prime \prime }-x_{0}\right){a^{\prime \prime }}_{13}+\left(y^{\prime \prime }-y_{0}\right){a^{\prime \prime }}_{23}-{a^{\prime \prime }}_{33\;}ck} \end{array}\right]$$

After substituting the third and fourth Equations from Equation (2) with the simplified equations (Table 1), the set of collinearity equations for the aerial (first image) and terrestrial (second image) imagery will look as follows, if φ”=90°:

(3) $$\left[\begin{array}{c} X=X_{0}^{\prime }+\left(Z-Z_{0}^{\prime }\right)\frac{\left(x^{\prime }-x_{0}\right){a^{\prime }}_{11}+\left(y^{\prime }-y_{0}\right){a^{\prime }}_{21}-{a^{\prime }}_{31\;}ck}{\left(x^{\prime }-x_{0}\right){a^{\prime }}_{13}+\left(y^{\prime }-y_{0}\right){a^{\prime }}_{23}-{a^{\prime }}_{33\;}ck}\\ Y=Y_{0}^{\prime }+\left(Z-Z_{0}^{\prime }\right)\frac{\left(x^{\prime }-x_{0}\right){a^{\prime }}_{12}+\left(y^{\prime }-y_{0}\right){a^{\prime }}_{22}-{a^{\prime }}_{32\;}ck}{\left(x^{\prime }-x_{0}\right){a^{\prime }}_{13}+\left(y^{\prime }-y_{0}\right){a^{\prime }}_{23}-{a^{\prime }}_{33\;}ck}\\ X=X_{0}^{\prime \prime }+\left(Z-Z_{0}^{\prime \prime }\right)\frac{-ck}{\left(x^{\prime \prime }-x_{0}\right){b^{\prime \prime }}_{13}+\left(y^{\prime \prime }-y_{0}\right){b^{\prime \prime }}_{23}}\\ Y=Y_{0}^{\prime \prime }+\left(Z-Z_{0}^{\prime \prime }\right)\frac{\left(x^{\prime \prime }-x_{0}\right){b^{\prime \prime }}_{12}+\left(y^{\prime \prime }-y_{0}\right){b^{\prime \prime }}_{22}}{\left(x^{\prime \prime }-x_{0}\right){b^{\prime \prime }}_{13}+\left(y^{\prime \prime }-y_{0}\right){b^{\prime \prime }}_{23}} \end{array}\right]$$

For ω” = 90° the set of equations will look as follows:

(4) $$\begin{array}{c} X=X_{0}^{\prime }+\left(Z-Z_{0}^{\prime }\right)\frac{\left(x^{\prime }-x_{0}\right){a^{\prime }}_{11}+\left(y^{\prime }-y_{0}\right){a^{\prime }}_{21}-{a^{\prime }}_{31\;}ck}{\left(x^{\prime }-x_{0}\right){a^{\prime }}_{13}+\left(y^{\prime }-y_{0}\right){a^{\prime }}_{23}-{a^{\prime }}_{33\;}ck}\\ Y=Y_{0}^{\prime }+\left(Z-Z_{0}^{\prime }\right)\frac{\left(x^{\prime }-x_{0}\right){a^{\prime }}_{12}+\left(y^{\prime }-y_{0}\right){a^{\prime }}_{22}-{a^{\prime }}_{32\;}ck}{\left(x^{\prime }-x_{0}\right){a^{\prime }}_{13}+\left(y^{\prime }-y_{0}\right){a^{\prime }}_{23}-{a^{\prime }}_{33\;}ck}\\ X=X_{0}^{\prime \prime }+\left(Z-Z_{0}^{\prime \prime }\right)\frac{\left(x^{\prime \prime }-x_{0}\right){c^{\prime \prime }}_{11}+\left(y^{\prime \prime }-y_{0}\right){c^{\prime \prime }}_{21}-{a^{\prime \prime }}_{31\;}ck}{\left(x^{\prime \prime }-x_{0}\right){c^{\prime \prime }}_{13}+\left(y^{\prime \prime }-y_{0}\right){c^{\prime \prime }}_{23}}\\ Y=Y_{0}^{\prime \prime }+\left(Z-Z_{0}^{\prime \prime }\right)\frac{\left(x^{\prime \prime }-x_{0}\right){c^{\prime \prime }}_{12}+\left(y^{\prime \prime }-y_{0}\right){c^{\prime \prime }}_{22}-{c^{\prime \prime }}_{32\;}ck}{\left(x^{\prime \prime }-x_{0}\right){c^{\prime \prime }}_{13}+\left(y^{\prime \prime }-y_{0}\right){c^{\prime \prime }}_{23}} \end{array}$$

where:

X, Y, Z—ground control point coordinates
X_0_^’^, Y_0_^’^, Z_0_^’^, ω’ φ’ κ’—exterior orientation parameters of aerial image
X_0_^’’^, Y_0_^’’^, Z_0_^’’^, ω^’’^, φ’’, κ’’—exterior orientation parameters of terrestrial image
x’, y’—image coordinates of a point on the aerial image
x’’, y’’—image coordinates of a point on the terrestrial image
c_k_, x_0_, y_0_—camera's interior orientation parameters
a’_11_, …, a’_33_—rotation matrix coefficients for the aerial image
a’’_11_, …, a’’_33_—rotation matrix coefficients for the terrestrial image
b’’_11_, …, b’’_33_—coefficients of the simplified rotation matrix for the terrestrial image when φ” = 90°
c’’_11_, …, c’’_33_—coefficients of the simplified rotation matrix for the terrestrial image when ω” = 90°.

The above Equations (3) and (4) can be rewritten in a simplified form:

(5) $$\left[\begin{array}{c} X={X^{\prime }}_{0}+\left(Z-{Z^{\prime }}_{0}\right)\;F\\ Y={Y^{\prime }}_{0}+\left(Z-{Z^{\prime }}_{0}\right)G\\ X={X^{\prime \prime }}_{0}+\left(Z-{Z^{\prime \prime }}_{0}\right)H\\ Y={Y^{\prime \prime }}_{0}+\left(Z-{Z^{\prime \prime }}_{0}\right)I \end{array}\right]$$

After transforming Equation (5), new equations are obtained for calculating the ground coordinates X, Y, Z of a point with known image-space coordinates:

(6) $$X=\frac{{X^{\prime \prime }}_{0}F-{Z^{\prime \prime }}_{0}FH-{X^{\prime }}_{0}H+{Z^{\prime }}_{0}FH}{F-H}\;Y=\frac{{Z^{\prime }}_{0}GI-{Y^{\prime }}_{0}I+{Y^{\prime \prime }}_{0}G-{Z^{\prime \prime }}_{0}GI}{G-I}\;Z=\frac{X-{X^{\prime }}_{0}}{F}+{Z^{\prime }}_{0}$$

In order to determine the ground coordinates XYZ, those equations for which XYZ can be calculated independently from other ground coordinates were selected. When the following are known: the interior orientation parameters of the camera: c_k_, x_0_, y_0_, the exterior orientation parameters of the video frame with the aerial orientation: X_0_^’^, Y_0_^’^, Z_0_^’^, ω’ ϕ’ κ’, and with the terrestrial orientation: X_0_^”^, Y_0_^”^, Z”, ω”, ϕ”, κ”, it is possible to calculate the ground coordinates of points, whose image coordinates had been measured on the video frames (x’, y’, x”, y”) based on the simplified collinearity equations.

Such a simplification of the collinearity equations is done to limit the amount of intermediate calculations when determining the coordinates of architectural structures in a ground coordinate system. It had therefore been decided, that the video camera's lens errors, such as radial and tangential distortion, which could increase the number of calculations, would not be taken into account in this prototype version of system.

Of course, the situation in which the difference in tilt angles of the camera during video registration is equal to 90° still remains only theoretical. The possibility of processing such a stereoimage was verified in a laboratory experiment. It was feared that because of the low coverage between the images and a large tilt angle, the absolute orientation of video images could be impossible.

## 3. Test Data Used in the Study

In order to verify these assumptions and check the possibility of conducting an absolute orientation of a pair of images for which the difference between tilt angle is near 90°, research was carried out on the test object in the shape of a 1 m × 2 m × 1 m cuboid. The test object was filmed with a Sony NEX-VG10 E video camera with Sony E 16 mm F2.8 fixed focal length lens [23] (Table 2). 

**Figure 2:**
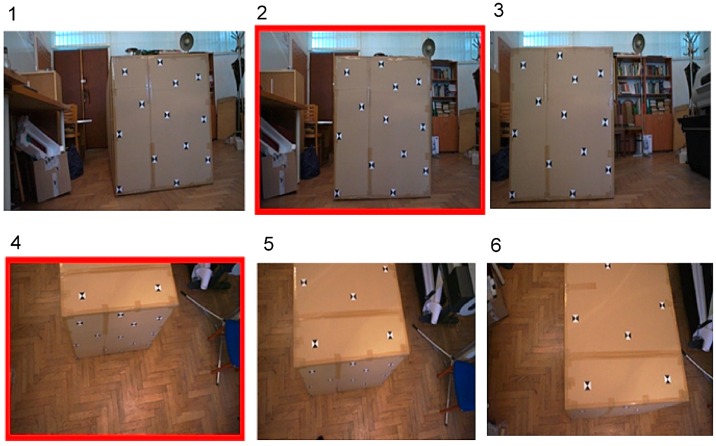
Test object video frames acquired from the simulated terrestrial level (frames **1**–**3**) and aerial level (frames **4**–**6**) Images number **2** and **4** are framed in red because for this pair of images, it is possible to conduct an absolute orientation when difference of tilt angle is near 90°.

A simulated UAV flight was performed to acquire the test object video data from the terrestrial and aerial level. Six video frames were selected from the videos: three with a terrestrial orientation and three with an aerial orientation (Figure 2).

Based on the acquired image data, the following processes were performed: (1) orientation of video frames from the terrestrial level, creating stereo: 1–2 and 2–3; (2) aerotriangulation of video frames from the UAV level, creating stereo: 10–11 and 11–12; (3) orientation of video frames acquired from terrestrial and UAV levels creating stereo 2–10. For the stereo 2–10 the difference in camera rotation angle about the Y axis was 74°. Table 3 presents values of angles of video frames' exterior orientation parameters. Values of angles have been calculated based on standard collinearity equations.

Table 4 below shows the base-distance ratio of each of the stereos as well as the spatial resolution in the XY plane (∆XY) of the geometric model and along the Z axis (∆Z) calculated from the following equations:

(7) $$\Delta XY=\frac{H}{ck}\delta \rho$$

(8) $$\Delta Z=\frac{H}{B}\Delta XY$$

where:

H—distance from the camera to the object
ck—camera/s focal length
δp—pixel size
B—base

For all the stereos, a planar resolution (ΔXY) of 1 mm was obtained. Moreover, the resolution (ΔZ) of the 2–10 stereo proved to be the lowest compared to other stereos because of the favourable base-to height ratio. Research conducted on the test object confirmed the validity of the assumption that it is possible to perform the orientation of video images acquired using a video camera mounted on a UAV.

## 4. Experiment on an Architectural Structure

**Figure 3:**
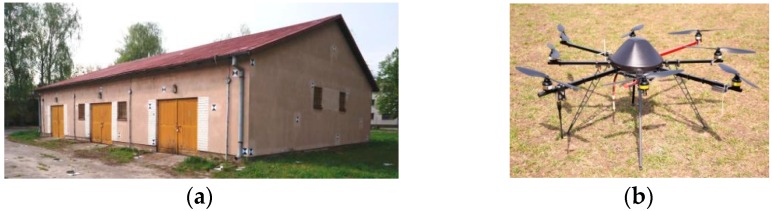
(**a**) Measured architectural structure; (**b**) UAV—octocopter used in studies.

Positive orientation results of video images obtained for the test object prompted the authors to investigate further. To this end, a photogrammetric flight of an unmanned mini-copter was performed over a building with a sloping roof and rectangular base with the following dimensions: 13 m × 20 m × 6 m (Figure 3).

### 4.1. Photogrammetric Flight Planning

In order obtain the video data, a Sony NEX-5N non-metric digital camera and Sony E 16 mm F2.8 lens were used. The camera weight with the lens was 336 g. The following factors were decisive in the choice of altitude: building dimensions, GSD, and terrestrial frame dimensions. The key equipment and flight plan parameters are listed in Table 5.

### 4.2. Choosing Targets for the Photogrammetric Network

**Figure 4:**
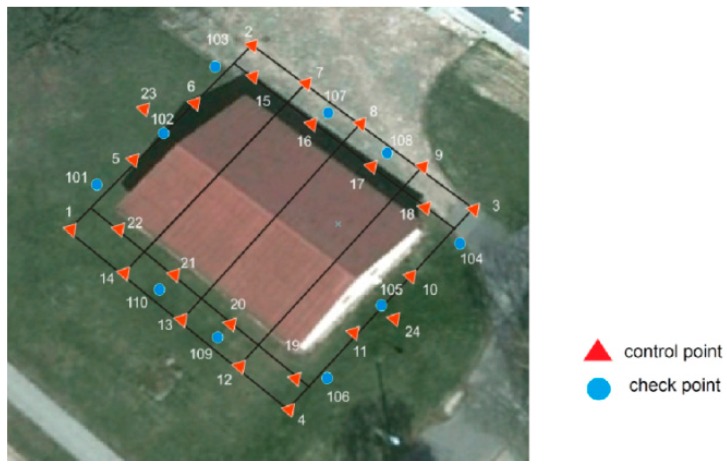
Photogrammetric terrestrial network around the building.

**Figure 5:**
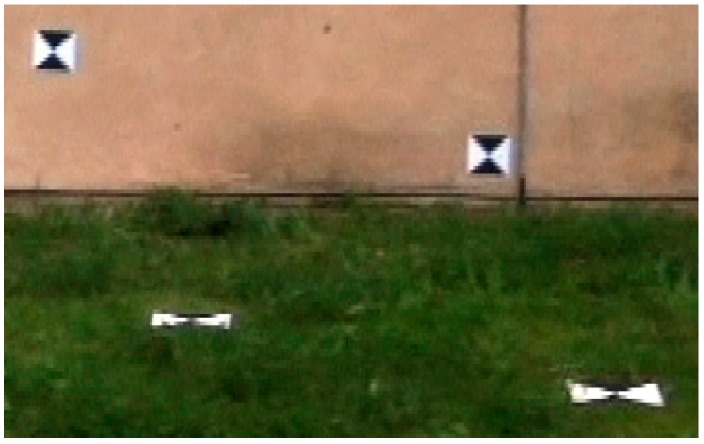
A part of a video image with targets placed on two surfaces: the building wall and on the ground.

Due to the low accuracy of the GPU-IMU systems in low-budget UAVs, it is recommended to use control points. In order to select the appropriate targets to act as control points, four groups of targets were designed: crosses, circles, squares and checkerboards. It was assumed that the targets for both aerial and terrestrial level applications should allow for clear identification of their centres, regardless of the distance and angle of imaging. At the testing stage, several possibilities for visualizing the GCP signals were examined. After considering both criteria, the checkerboard targets proved to be the best. A design was created for the placement of photogrammetric terrestrial network points on building walls and around the measured structure (Figure 4). The signals were made from a white board painted with black paint. The targets placed on the walls of the building were printed on A4 sheets, then laminated with non-reflective foil (Figure 5). The signals were made in two sizes: 30 cm × 30 cm (horizontal network) and 20 cm × 20 cm (points on the wall).

**Figure 6:**
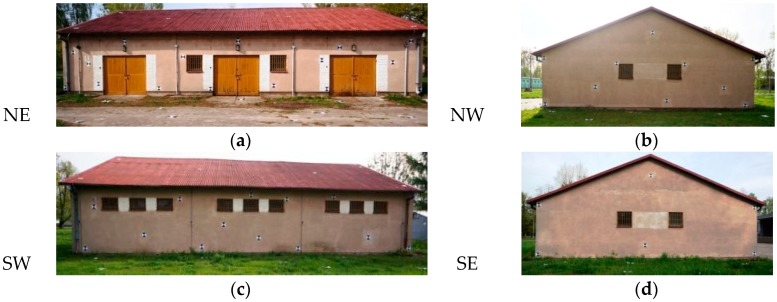
Photogrammetric network on the building walls and around the building. (**a**) Northeastern wall (NE); (**b**) Northwestern wall (NW); (**c**) Southwestern wall (SW); (**d**) Southestern wall (SE).

The terrestrial photogrammetric network consisted of 24 control points and 10 check points. Targets were also placed on the walls of the building—a total of 47 signals on the walls of the building and 12 signals on the roof (Figure 6). The coordinates of the points were measured using the Topcon Total Station with an error of ±6 mm.

**Figure 7:**
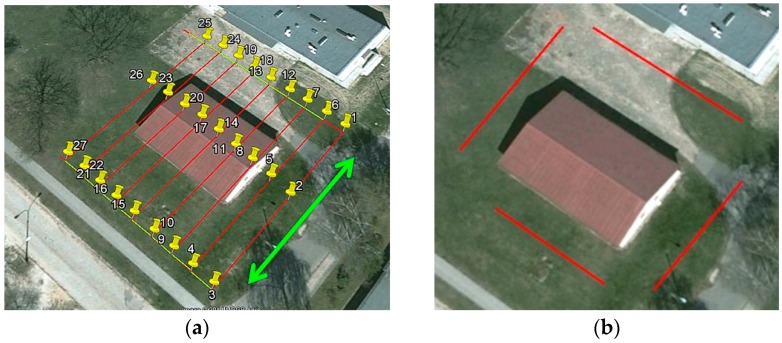
Flight plan for the aerial orientation (**a**) and terrestrial orientation (**b**). The numbers 1–27 represent the POIs. The red lines represent the UAV flight path, with the green line showing the direction of flight of the UAV.

Two different types of UAV flights were conducted. The first flight over the building, with the aerial orientation, was performed along 27 pre-designed POIs (Figure 7a). The second flight, with the terrestrial orientation, was performed along the building's four walls (Figure 7b). In this case the average flight altitude was 3 m above the ground level. For each wall, a set of two flights was performed at two different camera tilt angles.

**Figure 8:**
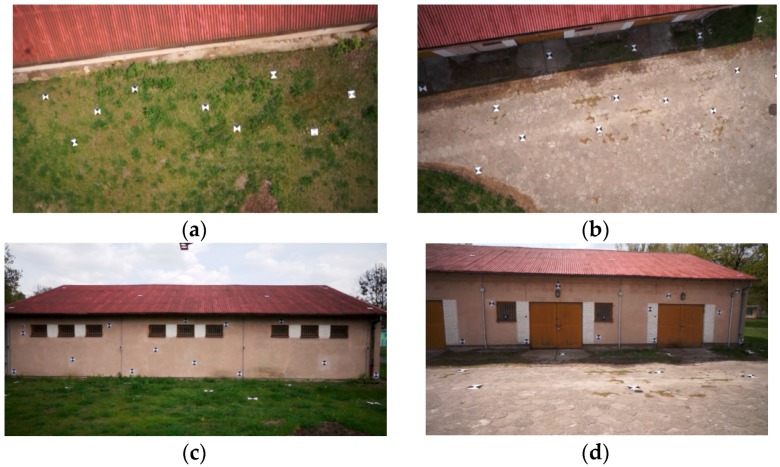
Example pairs of images from the terrestrial and aerial orientations: NE wall (**a**,**b**); SW wall (**c**,**d**).

Due to insufficient data (the targets were covered by vegetation), the south east (SE) wall of the building was not included further in this research. Pairs of images had been chosen from the acquired image sequences from both the aerial and terrestrial orientations to create stereopairs (Figure 8). The overlap area between images of two different orientations (aerial and terrestrial) includes the following features of the building: the basement, fragments of the side walls and the roof's edge.

### 4.3. Control Point and Tie Point Configurations

**Figure 9:**
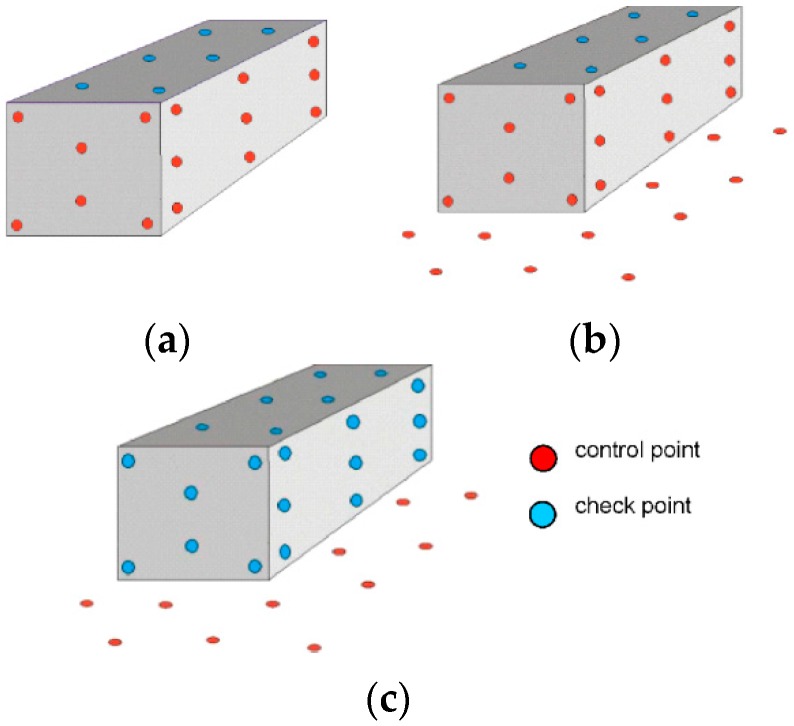
Three configurations of control points and tie points: (**a**) I—control points on the facade; (**b**) II—control points on the facade and around the object; (**c**) III—control points around the object.

Modern UAV systems are all equipped with a GPS/INS receiver, which records information about the location of the camera during acquisition. However, the precision of these systems is sometimes so low, that it is better to determine the exterior orientation parameters of an image using space resection, i.e., based on control point measurements. When performing measurements in field conditions, it is sometimes impossible to place measurement targets where they are most needed as the surface may be inaccessible (with very high structures) or it may simply be forbidden to do so (with cultural heritage structures). In the research work, three configurations of control points and tie points were considered: (a) control points on the facade (b) control points on the facade and around the object (c) control points around the object (Figure 9).

The exterior orientation parameters were determined from control point measurements, using three different configurations (Figure 9). When transitioning from the aerial orientation to the terrestrial orientation, the angle which changes by close to 90° will differ between the different walls. Namely, for the north eastern (NE) and south western (SW) walls it will be the φ angle, whereas for the north western (NW) wall it will be the ω angle.

Table 6 and Table 7 present values of angular exterior orientation and differences between the linear exterior orientation parameters. These values have been calculated based on standard collinearity equations.

The stereo orientation of video frames from two different levels was successful. It turned out to be impossible to perform an absolute orientation of aerial and terrestrial imagery with the control point distributed in accordance with configuration III—i.e., when control points are located only on the ground around the building. 

This research has shown that for a pair of images with an aerial and terrestrial exterior orientation and having an unfavourable configuration control points, it is impossible to obtain reliable values for the exterior orientation parameters calculated using only control points located around the building (configuration III).

When using the other two control point configurations (I—control points only on the walls and II—control points on the walls and around the building) similar values for the exterior orientation parameters were obtained (differences for the linear parameters of between 0–0.1 m and 0.5°–15° for the angular parameters).

## 5. Results of the Experiment

A verification was performed, whether it is possible to apply the simplified collinearity equations to determine the X, Y, Z coordinates of the building. Matlab software was used to run an algorithm for calculating the ground coordinates of points (X, Y, Z) based on classic equations sets Equation (2) and the simplified collinearity Equations (3) and (4). The process of determining coordinates used the following input data: known interior and exterior orientation parameters of the camera and the measured image coordinates of the points on the building's walls. The calculated coordinates were compared to theoretical values, which is shown in Table 8 below.

**Figure 10:**
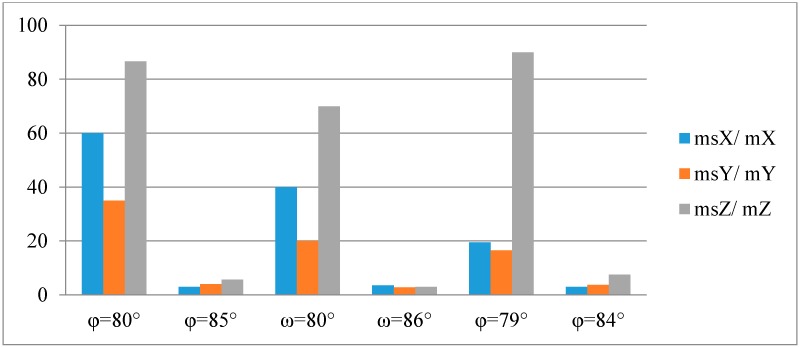
Ratios of the RMSE of the point coordinates calculated using simplified set of collinearity equations to the RMSE of the point coordinates calculated using classic collinearity equations for configuration I.

**Figure 11:**
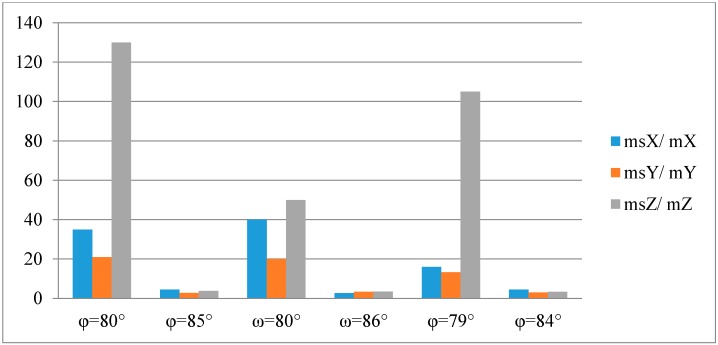
Ratios of the RMSE of the point coordinates calculated using simplified set of collinearity equations to the RMSE of the point coordinates calculated using classic collinearity equations for configuration II.

Figure 10 and Figure 11 below illustrate the ratios of the RMSE of the point coordinates (X, Y, Z) calculated using a classic set of collinearity equations to the RMSE of the point coordinates (X, Y, Z) calculated using the simplified collinearity equations.

Similar error values were obtained for all measured walls for tilt angles φ, ω ≈ 85°. This means that for tilt angles close to 90°, the mean errors of determining coordinates based on simplified collinearity equations are close to 10 cm. The closer the tilt angle is to 90°, the RMSE value increases for coordinates calculated using the classic set of equations.

An analysis of the results obtained using the simplified collinearity equations shows, that the worst results were achieved for a φ = 80° tilt angle. Slightly better results were obtained for the ω angle of the same value (80°). This means that when simplifying a collinearity equation using ω = 90°, the new collinearity equation is simplified by a smaller number of rotation matrix coefficients, than when the same is done with angle ϕ. It should be noticed, that when the tilt angle ϕ = 90°, 4 out of 5 rotation matrix coefficients are equal to zero (0). However, when the tilt angle ω = 90°, only one of these coefficients is equal to zero (0).

The greatest RMSE m_0Z_, both for the classic equation set and for the simplified collinearity equations, had been obtained for the Z coordinate. This is due to the fact that the Z coordinate is calculated based on the values of the other two coordinates (X and Y). Slightly higher errors had been observed for configuration II of the distribution of control and tie points. 

## 6. Determining the Relation between the Established and Actual Tilt Angle of the Video Camera

The research described above had shown that for tilt angles ϕ and ω close to 90°, the calculated ground coordinates of points on the building using the simplified set of equations are very close to the results obtained using the classic set of equations. This means that when performing a flight over a building using a copter, (in case of using the simplified equations) the tilt angle of the camera φ (ω) for all video frames should be within the 85°–90° range.

Therefore, for low-cost unmanned aerial copter systems, which do not have a flight stabilization system, it is essential to determine the difference between the established camera tilt angle ϕ (ω) defined during flight planning and the actual tilt angle value defined during the image orientation process. The difference between their values can differ for different copter systems. This difference depends on many factors, including the speed with which the platform is moving, it's mass, payload capacity, number of engines, etc. Most importantly, this difference in angles will also be affected by the speed and direction of winds. This difference can be determined empirically by conducting a series of test flights at different speeds.

In the article, an experiment had been conducted to show the change in the difference between the established and actual tilt angles of a camera mounted on a UAV computer system in relation to the speed of the platform. A series of 6 flights were performed at chosen speeds: 1, 3, 5, 7, 9, 11 m/s. The experiment was performed during a windless day.

**Figure 12:**
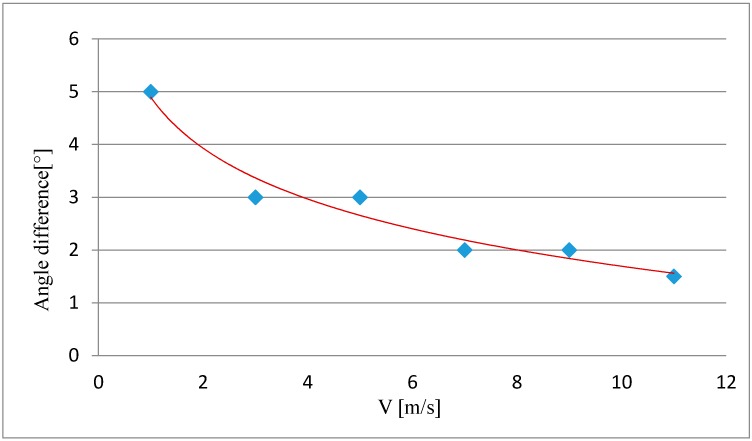
Chart of the relation between the difference in the established and actual camera tilt angles and the UAV speed.

For the UAV system flying at a speed of 3 m/s, the difference between the established camera tilt angle and the same angle derived from the exterior orientation parameters was equal to 3°. The experiment shows that with an increase in the UAV's speed, the difference between the established and actual tilt angles lessens, therefore making the platform's flight more stable (Figure 12). During video acquisition of an architectural structure at high flying speeds, the possibility of image blur must be taken into account. The assumption that the actual camera tilt angle ϕ (ω) is equal to the established angle is only possible with high-end copters, equipped with modern stabilization systems.

## 7. Discussion

Starting from the collinearity equations, which are the basis for photogrammetry, simplified mathematical model was proposed. The use of a simplified collinearity equations can significantly shorten the processing of image data from UAV. It was found that it is possible to perform an absolute orientation of video images acquired using a video camera mounted on a UAV, with a smooth transition from a terrestrial to aerial exterior orientation.

Experiments were conducted on a geometrically simple architectural structure. The video camera was mounted on a UAV copter system not equipped with a high precision GPS/INS measurement system. Therefore the exterior orientation parameters were determined using space resection—by measuring the location of control and tie points in two different distribution configurations. The target used to signalise these points made it possible to identify control points in two planes—on the wall and on the ground. The experiments have proven, that for two out of the three proposed control point distribution configurations—I and II (I—control points on the facade, II—control points on the facade and around the object, III—control points around the object) it is possible to obtain good results when processing video imagery. Slightly better results were obtained for configuration I.

The errors in point ground coordinates calculated using simplified collinearity equations oscillate about a few to a few hundred centimetres. When using the classic set of collinearity equations, the errors in coordinates are greater when the tilt angle ϕ (ω) ≈ 85°, i.e., when the value of this angle is close to the angle (90°). The opposite is true when the coordinates are calculate using the simplified collinearity equations, where the errors for all coordinates (m_0X_ m_0Y_, m_Z_) become smaller.

**Figure 13:**
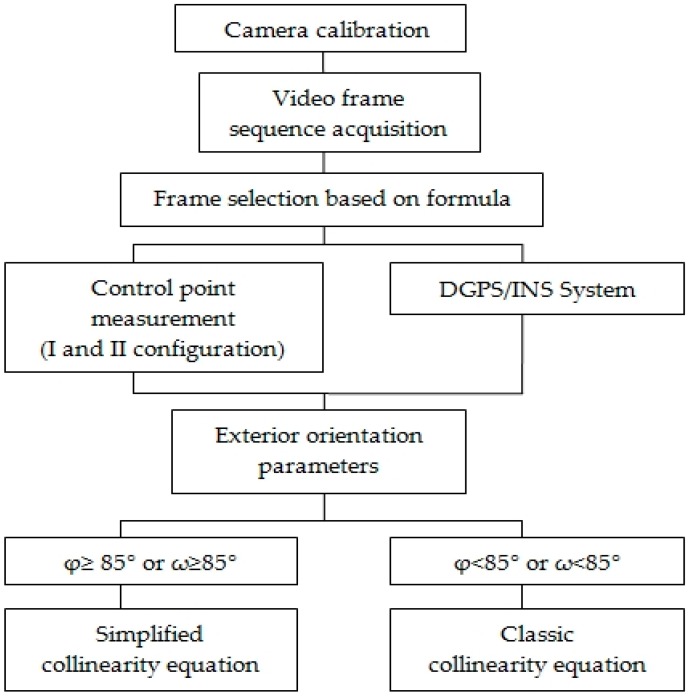
Proposed methodology for integrating video images acquired from terrestrial and aerial orientations of the camera mounted on the UAV.

The proposed method deals with the problem of integrating 3D models of the roof and walls of an architectural structure. The presented algorithm for determining the X, Y, Z coordinates of a structure had been established with the thought of designing an unmanned system, the aim of which would be to determine the coordinates on the surface of a structure in near-real-time. The designed algorithm could be implemented in a device onboard the UAV. Given known exterior orientation parameters, its purpose would be to automatically determine the X, Y, Z coordinates of points on the surface of a building. In the situation that one of the camera tilt angles ϕ or ω would be between 85°–90°, the set of simplified collinearity equations would be used to calculated the X, Y, Z coordinates of the points (Figure 13).

The proposed methods will shorten the calculation time by over 50%. In the case of a low cost system, the number of equations has a great influence on the computational speed.

## 8. Conclusions

This paper describes the issue of performing absolute orientation of video images which have two different exterior orientations of the camera mounted on the UAV: terrestrial and aerial. The proposed method can be used to determine the X, Y, Z coordinates of points based on a set of collinearity equations of a pair of images, with the equations for the terrestrial exterior orientation image being greatly simplified, assuming that the camera tilt angle is equal to 90°. The aim of this simplification is to limit the number of intermediate computations when calculating the coordinates of points on the surface of architectural structures in a ground coordinate system in the special case of image orientation mentioned.

## Figures and Tables

**Table 1:** Simplified rotation matrix coefficient equations when ϕ = 90° or ω = 90°.

Rotation Matrix Coefficient	Equation of Rotation Matrix Coefficient in the Classic Form (Rotation Sequence: ωφκ)	Simplified Equation of Rotation Matrix Coefficient for $\underset{\varphi \to 90{}^{\circ}}{\lim}f\left(\varphi \right)$	Simplified Equation of Rotation Matrix Coefficient for $\underset{\omega \to 90{}^{\circ}}{\lim}f\left(\omega \right)$
**a_11_**	cosφcosκ	0	cos φcosκ
**a_12_**	cosω sin κ+sinωsinφcosκ	cosωsinκ+sinωcosκ	sinφcosκ
**a_13_**	sinωsinκ−cosω sinφ cosκ	sinωsinκ−cosωcosκ	sinκ
**a_21_**	−cosφsinκ	0	−cosφsinκ
**a_22_**	cosωcosκ−sinωsinφsinκ	cosωcosκ−sinωsinκ	−sinφsinκ
**a_23_**	sinωcosκ+cosωsinφsinκ	cosωcosκ−sinωsinκ	cosκ
**a_31_**	sinφ	1	sinφ
**a_32_**	−sinωcosφ	0	−cosφ
**a_33_**	cosωcosφ	0	0

**Table 2:** The main parameters of the Sony Handycam NEX-VG10E video camera.

Camera	Sony Handycam NEX-VG10E
**Sensor size**	CMOS 23.4 mm × 15.6 mm
**Camera resolution**	1920 × 1080
**Pixel size**	10.8 μm
**Number of frames per second**	25 fps
**Video format**	AVCHD (MPEG-4 AVC (H.264))

**Table 3:** Angular exterior orientation parameters of the video frames of the test object.

Frame No.	φ (°)	ω (°)	κ (°)
1	−4.9	−90.3	95.0
2	−1.9	−92.2	90.7
3	−2.5	−92.6	90.3
10	6.1	−17.7	97.0
11	8.8	−20.1	96.6
12	5.5	−19.1	92.3

**Table 4:** Base-distance ratios and resolution of the stereo geometric model of test object.

Stereo	B (m)	H (m)	B/H	ΔXY (m)	ΔZ (m)
1–2	0.411	1.912	0.220	0.001	0.006
2–3	0.532	1.789	0.300	0.001	0.004
2–10	2.206	1.789	1.230	0.001	0.001
11–10	0.244	1.604	0.150	0.001	0.007
11–12	0.364	1.587	0.230	0.001	0.005

**Table 5:** Flight plan parameters.

Parameter	Value
Camera	Sony NEX-5N
Lens	Sony E 16 mm F2.8.
Height above terrain W	16 m
Scale denominator of a video frame M_z_	1000
Scale denominator of a video frame M_z_ for the roof	625
GSD roof/wall/terrain (mm)	3/5/7
Image swath	5.29 m × 9.41 m
Across base (q = 60%)	3.8 m

**Table 6:** Angular exterior orientation parameters calculated based on measuring control points distributed in different configurations.

Wall	Configuration	ω (°)		φ (°)		κ (°)	
		N	L	N	L	N	L
NE	I	−49.54	−3.59	−79.72	−3.34	−136.78	84.34
	II	−43.72	−2.92	−78.90	−2.58	−130.75	84.64
	III	-	-	-	-	-	-
NE	I	−46.23	−3.78	85.47	−4.67	−134.78	84.34
	II	−41.24	−2.71	−85.14	−2.24	−132.75	84.64
	III	-	-	-	-	-	-
NW	I	80.80	−11.72	3.26	−9.05	−3.52	77.20
	II	80.00	−11.55	3.29	−9.00	−3.51	77.27
	III	-	-	-	-	-	-
NW	I	86.04	−10.63	2.43	−8.12	−1.52	74.20
	II	86.34	−11.61	5.28	−9.42	−5.51	76.27
	III	-	-	-	-	-	-
SW	I	−70.04	−4.62	79.10	1.23	161.65	88.14
	II	−56.20	−5.95	79.49	−1.57	147.87	87.92
	III	-	-	-	-	-	-
SW	I	−71.67	−5.66	84.80	0.77	162.24	87.51
	II	−57.08	−5.58	83.69	−1.03	147.66	87.27
	III	-	-	-	-	-	-

**Table 7:** Differences between the linear exterior orientation parameters determined using different control point distribution configurations: I and II.

Configuration	Control Points			Check Points		
	dX (m)	dY (m)	dZ (m)	dX (m)	dY (m)	dZ (m)
I	0.005	0.008	0.003	0.014	0.014	0.005
II	0.010	0.014	0.018	0.037	0.046	0.016
III	-	-	-	-	-	-
I	0.007	0.007	0.007	0.016	0.004	0.009
II	0.014	0.008	0.018	0.017	0.003	0.028
III	-	-	-	-	-	-
I	0.009	0.007	0.004	0.019	0.027	0.096
II	0.015	0.016	0.015	0.003	0.012	0.001
III	-	-	-	-	-	-

**Table 8:** RMSE values of the point coordinates calculated using a simplified set of collinearity equations and RMSE values of the point coordinates calculated using the standard collinearity equations for two variants of distribution of the control and tie points.

Tilt Angle	Configuration	mX (cm)	mY (cm)	mZ (cm)	msX (cm)	msY (cm)	msZ (cm)
**NE φ = 80°**	I	1	1	3	60	35	260
	II	2	2	2	70	42	260
**NE φ = 85°**	I	2	2	3	6	8	17
	II	2	4	4	9	11	15
**NW ω = 80°**	I	1	1	2	40	20	140
	II	1	1	3	40	20	150
**NW ω = 86°**	I	2	4	4	7	11	12
	II	3	3	4	8	10	14
**SW φ = 79°**	I	2	2	2	39	33	180
	II	2	3	2	32	40	210
**SW φ = 84°**	I	2	3	2	6	11	15
	II	2	4	5	9	12	17

mX—RMSE of the X coordinate calculated using the classic set of collinearity equations; mY—RMSE of the Y coordinate calculated using the classic set of collinearity equations; mZ—RMSE of the Z coordinate calculated using the classic set of collinearity equations; msX—RMSE of the X coordinate calculated using the simplified set of collinearity equations; msY—RMSE of the Y coordinate calculated using the simplified set of collinearity equations; msZ—RMSE of the Z coordinate calculated using the simplified set of collinearity equations.
